# Comparison of Metabolic Control in Children and Adolescents Treated with Insulin Pumps

**DOI:** 10.3390/children11070839

**Published:** 2024-07-10

**Authors:** Agnieszka Lejk, Karolina Myśliwiec, Arkadiusz Michalak, Barbara Pernak, Wojciech Fendler, Małgorzata Myśliwiec

**Affiliations:** 1 Department of Pediatrics, Diabetology and Endocrinology, Medical University of Gdansk, 80-210 Gdansk, Poland; agnieszkalejk@gumed.edu.pl (A.L.);; 2 Department of Biostatistics and Translational Medicine, Medical University of Lodz, 92-215 Lodz, Poland; 3 Department of Pediatrics, Diabetology, Endocrinology and Nephrology, Medical University of Lodz, 91-738 Lodz, Poland

**Keywords:** insulin pump, closed loop, predictive suspend

## Abstract

Background: While insulin pumps remain the most common form of therapy for youths with type 1 diabetes (T1DM), they differ in the extent to which they utilize data from continuous glucose monitoring (CGM) and automate insulin delivery. Methods: The aim of the study was to compare metabolic control in patients using different models of insulin pumps. This retrospective single-center study randomly sampled 30 patients for each of the following treatments: Medtronic 720G without PLGS (predictive low glucose suspend), Medtronic 640G or 740G with PLGS and Medtronic 780G. In the whole study group, we used CGM systems to assess patients’ metabolic control, and we collected lipid profiles. In three groups of patients, we utilized CGM sensors (Guardian 3, Guardian 4, Libre 2 and Dexcom G6) to measure the following glycemic variability proxy values: time in range (TIR), time below 70 mg/dL (TBR), time above 180 mg/dL (TAR), coefficient of variation (CV) and mean sensor glucose. Results: Medtronic 640G or 740G and 780G users were more likely to achieve a target time in the target range 70–180 mg/dL (≥80%) [Medtronic 720G = 4 users (13.3%) vs. Medtronic 640G/740G = 10 users (33.3%) vs. Medtronic 780G = 13 users (43.3%); *p* = 0.0357)] or low glucose variability [Medtronic 720G = 9 users (30%) vs. Medtronic 640G/740G = 18 users (60%) vs. Medtronic 780G = 19 users (63.3%); *p* = 0.0175)]. Conclusions: Any integration between the insulin pump and CGM was associated with better glycemic control. More advanced technologies and artificial intelligence in diabetes help patients maintain better glycemia by eliminating various factors affecting postprandial glycemia.

## 1. Introduction

Type 1 diabetes (T1DM) is the most common disorder of glucose metabolism in children and adolescents aged 5 to 18 years regardless of socioeconomic and educational status. It is treated with functional intensive insulin therapy which aims to supplement near-physiological amounts of exogenous insulin subcutaneously. This is achieved by using multiple daily injections or an insulin pump. According to the clinical recommendations of the Polish Diabetes Association, the children and adolescents with T1DM should aim for an HbA1c ≤ 6.5% (≤48 mmol/L) and a TIR > 70% [1]. Achieving these targets can be challenging due to the need for intensive self-management, including day planning, carbohydrate counting and making multiple decisions based on blood glucose levels. Most of the patients have trouble maintaining such rigor in everyday life. At the very beginning, right after the diagnosis of the disease, they try to follow the rules learned during their education in the hospital. Unfortunately, after a few years, there starts to be a problem with remembering about insulin before meals, and there are even situations where the insulin is delivered when the glycemia starts to be above the range checking out from the sensor. Personality and self-esteem are the most important features influencing further metabolic control of diabetes [2]. In many publications, authors emphasize even such factors as sex, age, illness type and perceptions of self in coping with chronic disease. Considering the longer observation, the problem with acceptance can start independently from the length of disease, personality or intolerance by society [3]. However, the latest technology makes the life of the patients easier. The standard of clinical care is continuous glucose monitoring (CGM) for every patient irrespective of the used method of insulin administration. In addition to therapy management, CGM provides new ways of assessing glycemic control. One such metric is time in target range (TIR), defined as time per day spent in the glucose range between 70 and 180 mg/dL. It provides a measure of dispersion and can distinguish between individuals with high and low glucose variability, even if their HbA1c values are similar [4]. Patients should aim for at least 80%. In addition to time in range, the next parameter is the coefficient of variation for glucose (CV), representing overall glycemic variability. The therapeutic target for CV is ≤36% [5]. Other metrics of metabolic control are time below range (TBR), defined as time per day spent in the glucose range below 70 mg/dL, and time above range (TAR), defined as time spent in the glucose range above 180 mg/dL [6]. The target for TBR is less than 4% and, for TAR, not more than 25%.

In Poland, the most common treatment for children and adolescents is an insulin pump. The devices differ from each other in terms of the connection between the insulin pump and CGM. Currently, the patient can use the system without predictive low glucose suspend (PLGS), with PLGS or with an advanced closed-loop hybrid system (AHCL) [7]. The systems without any connection between CGM and the insulin pump can be represented by the Medtronic 720G. It has some limitations, such as a lack of automation in order to reduce the basal insulin level when it comes to hypoglycemia [8]. PLGS, represented by the Medtronic 640G or 740G series, stops insulin administration on its own and resumes when the blood glucose level is in a safe range. It has been proved to be very effective in the prevention of hypoglycemia [9]. 

In 2020, an advanced closed-loop hybrid system (AHCL) was approved in Europe. This device has made it possible to optimize the metabolic balance by adjusting insulin doses between meals and overnight without patient involvement [10]. The advanced SmartGuard algorithm in this system automates and personalizes the delivery of basal insulin, adjusting it around the clock, thereby correcting high glucose values and protecting against low glucose values. Studies show that the use of the AHCL significantly improves glycemic control in patients with T1DM, mainly by increasing the time in tight range (TITR) [11]. 

Nowadays, in diabetology, the role of a proper quality of life is emphasized a lot. Therefore, the closed-loop systems have started to be very popular. Children and adolescents look for the treatment which helps them obtain the life most similar to that of their peers. There are at least 40 factors that influence the level of glycemia, not only regular meals or the correct counting of the grams of carbohydrates. Stress and infection can influence the patients’ glycemia. That could be because they do not achieve satisfactory metabolic control even though they try hard. An AHCL significantly increases the quality of life compared to insulin pumps without these functions. Additionally, the AHCL reduces the frequency of patients’ interference in insulin therapy, which is why they have a chance of having more comfort in life as well as improved glycemia during the whole day [12]. Insulin delivery is much simpler, so the patients must focus only on properly counting the grams of carbohydrates and leading a healthy lifestyle [13]. However, a healthy lifestyle in many cases is hard to achieve. If the children from the beginning had improper habits such as eating a lot of simple sugar from sweets, cereals, dairy products or beverages, it is hard to change. The same issue applies to regular activity instead of a sedentary lifestyle [14]. Currently, since the COVID-19 pandemic, a lot of children avoid activity after school by having excuses. Even more frequently, the parents choose homeschooled education for their children instead of sending them to school, which causes even less activity and more problems with obtaining a healthy lifestyle.

According to the previous paragraph, in which we emphasized the advantages of the AHCL, a need arises to compare the closed-loop pumps with systems with PLGS and without PLGS due to ambiguous research results in this area. The aim of our study was to compare metabolic control in patients with T1DM using the Medtronic 720G, Medtronic 640G/740G and Medtronic 780G. The essence of our study was to confirm that only integrated systems will allow patients to be safe from hypoglycemia, prevent acute and chronic complications and ensure the quality of life is the same as that of healthy individuals.

## 2. Materials and Methods

### 2.1. Participants, Recruitment and Study Design

This was a retrospective, observational single-center study. We recruited pediatric patients with diagnosed T1DM according to the criteria of the International Society for Pediatric and Adolescent Diabetes (ISPAD) guidelines [15]. All the patients were under the care of the Clinic of Pediatrics, Diabetology and Endocrinology at the University Clinical Center of Gdańsk, Poland. The patients from the study group had average or high socioeconomic and educational status. Currently, the clinic cares for 700 patients with type 1 diabetes. Of the patients, 70% are treated with a personal insulin pump, and, therefore, 30% are treated with multiple daily injections by pen. Most patients are between 10 and 16 years of age with an average glycated hemoglobin level of 7.2%. The study group is representative of the clinic’s patients because their average age range is identical to the average age of the clinic’s patients. Among those, we randomly selected 30 children per pump category for analysis (Medtronic 720G without PLGS (predictive low glucose suspend), Medtronic 640G or 740G with PLGS, Medtronic 780G). The study group was characterized by age, weight, height and duration of diabetes type 1. All data were collected from on-site medical documentation or (for CGM data) online cloud accounts such as CareLink Professional software, LibreView and Dexcom Clarity between November 2023 and February 2024. The anthropometric parameters such as body mass and height were collected during the visit to the diabetes clinic. We calculated the body mass index (BMI) z-score for every patient. Our results referred to the World Health Organization (WHO) reference values [16].

### 2.2. Ethics Statement

The study protocol was approved by the Bioethical Commission of the Medical University of Gdansk (no. KB/313/2024).

### 2.3. Collecting Clinical and Continuous Glucose Monitoring Data

In the entire study group, the lipid profiles were collected during the observation period. Additionally, we used CGM systems to assess patients’ metabolic control. In three groups of patients, we utilized CGM sensors (Guardian 3, Guardian 4, Libre 2 and Dexcom G6) to measure glycemic variability proxy values: time in range (TIR), time below 70 mg/dl (TBR), time above 180 mg/dL (TAR), coefficient of variation (CV) and mean sensor glucose.

### 2.4. Statistical Analysis

Continuous variables are summarized as medians and 25–75% ranges. Between-groups comparisons were performed with Kruskal–Wallis test and appropriate post hoc tests. The rates of meeting diabetes therapeutic targets were compared using global chi-square following by 2 × 2 post hoc chi-square tests under Bonferroni correction. All statistical analyses were performed in STATISTICA 13.1 (Dell Inc., Round Rock, TX, USA). The *p* value was considered to be significant at <0.05. No correction for multiple comparisons was applied.

## 3. Results

### 3.1. Study Group Characteristics

A total of 90 patients were enrolled in the study [30 (33.3%) per pump technology, 53 (58.9%) boys with a median age of 12.9 (11–15) years and 5.1 (2.2 to 8.4) years]. The total dose of insulin of patients was 0.8 (0.7–1) units/kg, and BMI (body mass index) was in the 58th (35–79) percentile. All patients were Caucasian, and, on clinical evaluation, reported only T1DM, with no signs of malnutrition. The detailed clinical characteristics of the group are presented in Table 1. All technology-based subgroups presented a similar sex structure [no. (%) of females for Medtronic 720G = 13 (43.3%), for Medtronic 640G/740G = 14 (46.7%) and for Medtronic 780G = 10 (33.3%), *p* = 0.5543].

Children using the Medtronic 720G had the longest T1DM duration [7 (5.2 to 9.1) years]. Those using the Medtronic 640/740G presented the lowest insulin requirements [0.7 (0.5 to 0.9) UI/kg, *p* < 0.05 vs. other pumps] but also the lowest time of sensor use [85 (64 to 91)% of expected data available]. The detailed comparisons are presented in Table 1.

### 3.2. Results of Diabetes Control for Different Technologies

**Figure 1:**
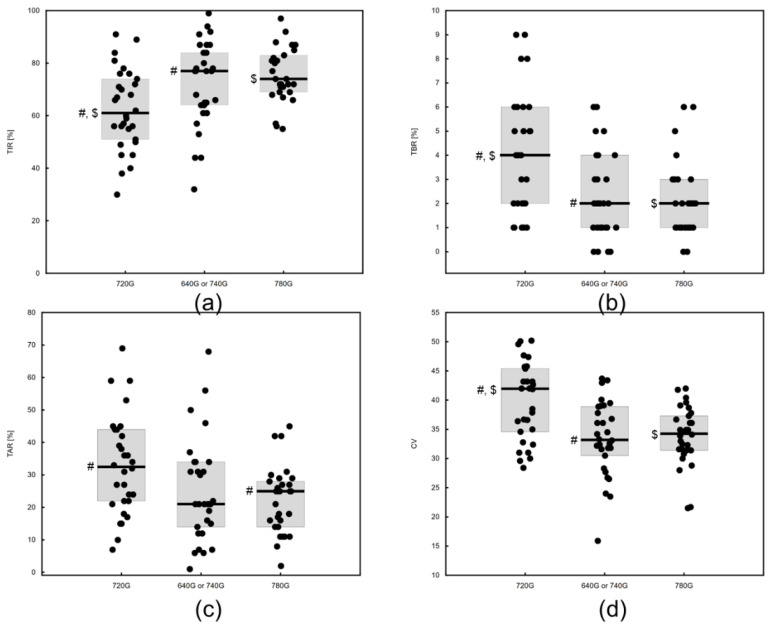
(**a**) Time in range for 720G, 640G/740G and 780G; (**b**) time below range for 720G, 640G/740G and 780G; (**c**) time above range for 720G, 640G/740G and 780G; (**d**) coefficient of variation for glucose for 720G, 640G/740G and 780G. #, $ denote significant (*p* < 0.05) pair-wise comparisons in post hoc tests.

The groups differed in TIR (Medtronic 720G: 61 (51 to 74)%, Medtronic 640/740G: 77 (64 to 84)%, Medtronic 780G: 74 (69 to 83)%, *p* = 0.0025), as well as hypoglycemia [TBR—Medtronic 720G: 2 to 6%, Medtronic 640/740G: 2 (1 to 4)%, Medtronic 780G: 2 (1 to 3)%, *p* = 0.0025] and hyperglycemia metrics [TAR—Medtronic 720G: 32.5 (22 to 44)%, Medtronic 640/740G: 21 (14 to 34)%, Medtronic 780G: 25 (14 to 28)%, *p* = 0.0141]. Glycemic variability was also highest in those treated with the Medtronic 720G [CV: 41.95 (34.6 to 45.4)%, Medtronic 640/740G: 33.2 (30.5 to 38.9)%, Medtronic 780G: 34.3 (31.4 to 37.3)%, *p* = 0.0010]. A graphical representation and post hoc comparisons are included in Figure 1.

Those treated with the Medtronic 780G most often achieved the target TIR ≥ 80% [13 (43.3)%], followed by those treated with the Medtronic 640/740G [10 (33.3)%, *p* = 0.2980 vs. Medtronic 780G) or the Medtronic 720G [4 (13.3)%, *p* = 0.0102 vs. 780G]. The target for CV < 36% was met similarly by 19 (63.3%) of Medtronic 780G users, 18 (60%) of Medtronic 640/740G users (*p* > 0.9999 vs. Medtronic 780G) and 9 (30%) of Medtronic 720G users (*p* = 0.0199 vs. Medtronic 780G).

There were no significant differences between the model of insulin pump and lipid profile (Table 2).

## 4. Discussion

The results of our study reveal a few interesting observations. Firstly, stable glycemic control in patients may significantly differ depending on the model of insulin pump and on whether the pump has a system without PLGS (predictive low glucose suspend), with PLGS or with an advanced closed-loop hybrid system (AHCL). “The most advanced treatment using ACHL system was reported to influence more stable glycemic control and improve the quality of life not only children and adolescence with T1DM, but the whole family”. The success of these systems and a significantly higher time in range have been observed in many publications comparing different models of insulin pumps [17]. However, in our study, we did not observe such results because of the size of study group. In our future studies, we propose to not only increase the size of the group of patients, but also divide it into subgroups according to gender, age and duration of treatment with a given method. That could make our results more reliable. Additively, we could consider also dividing the patient group into those who follow the minimal requirements considering daily habits, proper counting, etc., and those who do not wonder about the consequences of their lifestyle choices.

According to the results of our study, patients treated with a closed-loop hybrid system most often achieved the target TIR ≥ 80%. Therefore, the current results are the same as those of most of the publications connected with that theme, in which the advantages of the AHCL are emphasized [18]. However, the highest results of TAR, TBR and CV were achieved by patients on insulin pumps without any connection between the devices. Our observation is consistent with many studies on this topic [19]. Despite the AHCL, not all patients have stable glycemic control. The main reason is that many children and adolescents with T1DM do not follow the minimal requirements such as using a bolus calculator, properly counting the grams of carbohydrates and even having appropriate nutritional habits [20]. The same issue was observed in our study. Even though patients know everything about a low glycemic diet, regular meals and drinking a lot of water, they do not put these into practice. In our observation, achieving a proper lifestyle, including proper nutrition and activity, is one of the hardest things to follow.

However, there was a large percentage of patients treated by PLGS who achieved the same good metabolic control. Moreover, we noticed that patients on a PLGS system used the sensor less even though they had high time in range. In many cases, the sensor was disconnected. However, in many publications, one can read about the same issue, where patients do not have the chance to fully use the value of their insulin pump [21]. The main problem is that, in many cases, even the parents do not understand that they are supposed to check if their children know how to use the system or if it is connected properly. This situation often occurs when their children have had diabetes for a long time. It starts to be a common routine for them so they do not care as much as they did at the beginning of disease [22].

Additively, the AHCL improves the quality of life for all users. The algorithm gives automated bolus insulin corrections to combat hyperglycemia, as well as building low-glucose protection [23]. Therefore, the best metabolic control of the disease is supposed to be with those systems. Moreover, the study’s strength is that we compare the insulin pumps with a connection between the sensor and insulin pump, those without any connection and the one with the advanced closed-loop hybrid system. These kinds of studies give us a full perspective and highlight the advantages of closed-loop pumps as well as any kind of connection between the insulin pump and sensor.

Furthermore, even though we have mentioned that it is possible to achieve comparable metabolic balance with different models of insulin pumps, the latest publications emphasize the advantage of AHCLs compared to those with or without a PLGS system [24]. The safety and efficacy of AHCLs are seen even in children younger than 6 years old [25]. Even in a large meta-analysis from 2024, it is concluded that AHCLs significantly improve glycemic control, reducing the tendency towards to hypoglycemia and hyperglycemia and maintaining a higher TIR. The systematic review included 24 randomized trials with every AHCL available worldwide [26].

To sum up, our study requires more complex future observation. Many publications show only the advantages of closed-loop pumps, which were not emphasized in our results [27]. Many patients report the satisfaction of using this kind of system in everyday life. There is proof that day and night glycemic control is much better with closed-loop pumps; that is why our aim is to continue our studies in the nearest future.

### Strengths and Limitations

The limitations of our study are the size of the study group and the single-center nature of the study. Additionally, attention should be paid to other work limitations, i.e., age of onset of the disease, duration of the disease and duration of treatment with pump therapy. Numerous publications indicate that the longer the disease duration, the worse the metabolic control observed in continuous glucose monitoring systems [28]. The reason why it is happening so often is that patients and their parents are tired of continuous control. Everything the patients will do depends on the glycemia they have. The main issue starts to be when the patient is trying hard yet still their glycemia is not as stable as it is supposed to be if they want to go out with friends or eat what they want whenever they want. Secondly, our limited observation, giving only parameters from the three months, does not emphasize the advantages of closed-loop pumps. It could be limitation of our study, and longer prospective studies are required to fully compare metabolic control in patients using different models of insulin pumps. On the other hand, the longer the duration of pump therapy, the worse glycemic control the patients have [29]. In that case, it relates to people’s psychology. A new device causes patients’ involvement in keeping a time in range that is as good as possible by maintaining healthy habits and the rules of counting and giving the bolus of insulin correctly.

## 5. Conclusions

We observed that any kind of connection between the insulin pump and continuous glucose monitoring can give patients better metabolic control of their disease. The patients without any connection had worse results in TIR, TAR, TBR and CV. More advanced technologies and artificial intelligence in diabetes help patients maintain better glycemia by eliminating various factors affecting postprandial glycemia. Adjustment of the insulin doses by the device between meals makes it easier to cope with chronic disease. Our research has proven that combining a pump with a sensor allows children and adolescents with T1DM to maintain better parameters of metabolic control.

## Figures and Tables

**Table 1:** Clinical characteristics of users of different pumps. NA—not applicable.

Group Indices	Medtronic 720G without PLGS	Medtronic 640G or 740G with PLGS	Medtronic 780G	*p* Value
Continuous	Median (25 to 75%)	Median (25 to 75%)	Median (25 to 75%)	
Age [years]	15 (12 to 16) ^#,$^	13 (11 to 14) ^#^	12 (10 to 14) ^$^	**0.0090**
T1DM duration [years]	7 (5.2 to 9.1) ^#^	2.3 (1.2 to 7) ^#^	3.9 (2.3 to 7.5)	**0.0019**
DDI (daily dose of insulin) [units/kg]	0.8 (0.8 to 1) ^#^	0.7 (0.5 to 0.9) ^#,$^	0.9 (0.7 to 1.1) ^$^	**0.0031**
Weight [kg] z-score percentile	57.1 (47 to 66.8) 0.4 (−0.1 to 0.9) 64 (47 to 81)	44.7 (37.5 to 62.3) 0.3 (−0.6 to 0.9) 60.5 (27 to 81)	47.8 (33.3 to 54) 0.1 (−0.5 to 0.6) 54.5 (31 to 73)	NA 0.3494 NA
Height [cm] z-score percentile	166.7 (157 to 179) 0.4 (−0.6 to 1) 64.5 (29 to 84)	156.3 (146 to 165.8) 0.1 (−0.7 to 1) 54 (26 to 84)	153.3 (141.8 to 167) 0.1 (−0.8 to 0.7) 53 (20 to 76)	NA 0.3103 NA
BMI [kg/m^2^] z-score percentile	20.2 (18.5 to 21.8) 0.3 (−0.3 to 0.7) 61 (37 to 76)	19.3 (16.6 to 21.5) 0.4 (−0.4 to 0.8) 64.5 (34 to 79)	18.3 (16.8 to 20.8) 0.1 (−0.3 to 0.5) 56 (38 to 69)	NA 0.7838 NA
CSII (continuous subcutaneous insulin infusion) duration [years]	5.4 (4.2 to 7.6) ^#^	1.5 (1 to −2) ^#^	3.5 (1 to 6.7)	**0.0039**
Duration of present insulin pump therapy [years]	0.9 (0.5 to 2.7)	1.3 (0.7 to 2)	1.4 (1 to 2.1)	0.2160
Time of sensor use [%]	91.5 (88 to 97) ^#,$^	85 (64 to 91) ^#^	96 (95 to 98) ^$^	**<0.0001**
Time of SmartGuard use [%]	NA	NA	97.5 (95 to 99)	NA

Post hoc chi-square tests under Bonferroni correction. ^#^, ^$^ denote significant (*p* < 0.05) pair-wise comparisons in post-hoc tests. The bolded values have a statistical meaning.

**Table 2:** Lipid profile compared between users of different pumps.

Group Indices	Medtronic 720G without PLGS	Medtronic 640G or 740G with PLGS	Medtronic 780G	*p* Value
Continuous	Median (25 to 75%)	Median (25 to 75%)	Median (25 to 75%)	
TC [mg/dL]	161.5 (150 to 175)	170 (157 to 192)	170.5 (152 to 194)	0.1537
LDL [mg/dL]	89 (78 to 104)	98.5 (90 to 113)	91 (84 to 111)	0.1937
HDL [mg/dL]	57 (50 to 68)	61.5 (45 to 71)	62 (49 to 69)	0.8801
TG [mg/dL]	61.5 (44 to 72)	59.5 (47 to 76)	74.5 (47 to 84)	0.5567

Post hoc chi-square tests under Bonferroni correction.

## Data Availability

Data presented in this study are available on request from the corresponding author. The data are not publicly available due to subjects’ privacy.
